# Coarse-Grained Simulations of Topology-Dependent Mechanisms of Protein Unfolding and Translocation Mediated by ClpY ATPase Nanomachines

**DOI:** 10.1371/journal.pcbi.1004675

**Published:** 2016-01-06

**Authors:** Andrea N. Kravats, Sam Tonddast-Navaei, George Stan

**Affiliations:** Department of Chemistry, University of Cincinnati, Cincinnati, Ohio, United States of America; University of North Texas System College of Pharmacy, UNITED STATES

## Abstract

Clp ATPases are powerful ring shaped nanomachines which participate in the degradation pathway of the protein quality control system, coupling the energy from ATP hydrolysis to threading substrate proteins (SP) through their narrow central pore. Repetitive cycles of sequential intra-ring ATP hydrolysis events induce axial excursions of diaphragm-forming central pore loops that effect the application of mechanical forces onto SPs to promote unfolding and translocation. We perform Langevin dynamics simulations of a coarse-grained model of the ClpY ATPase-SP system to elucidate the molecular details of unfolding and translocation of an *α*/*β* model protein. We contrast this mechanism with our previous studies which used an all-*α* SP. We find conserved aspects of unfolding and translocation mechanisms by allosteric ClpY, including unfolding initiated at the tagged C-terminus and translocation via a power stroke mechanism. Topology-specific aspects include the time scales, the rate limiting steps in the degradation pathway, the effect of force directionality, and the translocase efficacy. Mechanisms of ClpY-assisted unfolding and translocation are distinct from those resulting from non-allosteric mechanical pulling. Bulk unfolding simulations, which mimic Atomic Force Microscopy-type pulling, reveal multiple unfolding pathways initiated at the C-terminus, N-terminus, or simultaneously from both termini. In a non-allosteric ClpY ATPase pore, mechanical pulling with constant velocity yields larger effective forces for SP unfolding, while pulling with constant force results in simultaneous unfolding and translocation.

## Author Summary

Cell survival is critically dependent on tightly regulated protein quality control, which includes chaperone-mediated folding and degradation. In the degradation pathway, AAA+ nanomachines, such as bacterial Clp proteases, use ATP-driven mechanisms to mechanically unfold, translocate, and destroy excess or defective proteins. Understanding these remodeling mechanisms is of central importance for deciphering the details of essential cellular processes. We perform coarse-grained computer simulations to extensively probe the effect of substrate protein topology on unfolding and translocation actions of the ClpY ATPase nanomachine. We find that, independent of SP topology, unfolding proceeds from the tagged C-terminus, which is engaged by the ATPase, and translocation involves coordinated steps. Topology-specific aspects include more complex unfolding and translocation pathways of the *α*/*β* SP compared with the all-*α* SP due to high stability of *β*-hairpins and interplay of tertiary contacts. In addition, directionality of the mechanical force applied by the Clp ATPase gives rise to distinct unfolding pathways.

## Introduction

Protein quality control mechanisms, which include folding assistance or degradation of abnormal proteins, are critical for maintaining cell viability and function and for preventing protein aggregation pathways that underlie neurodegenerative diseases. In bacteria and eukaryotic organelles, protein degradation and disaggregation are performed by Caseinolytic proteases (Clp), which are self-compartmentalized molecular machines comprising ATPase and peptidase components. Clp ATPases are members of the AAA+ (ATPases Associated with various cellular Activities) superfamily [1, 2] that performs DNA replication, microtubule severing, transporting cargo along microtubules, protein unfolding and translocation, and disaggregation [2–4]. Structurally, AAA+ machines are oligomeric ring assemblies with monomers that generate catalytic activity through one or two conserved AAA domains [5, 6]. Crystal structures [7–13] and electron microscopy images [14, 15] revealed that Clp ATPases have a homo-hexameric single-ring (ClpX, ClpY/HslU) or double-ring (ClpA, ClpB) structure which encloses a central channel with a diameter of ∼20–30 Å at its entrance and a width of ∼10–20 Å at the narrowest point. The peptidase component (ClpP or ClpQ), which is responsible for the proteolytic action, associates coaxially with one or two ATPase particles [5, 7, 16–18]. ClpP forms complexes with ClpA or ClpX, whereas ClpQ (HslV) binds ClpY (HslU). Due to the narrow openings within the ATPase channel, substrate proteins (SP) must be unfolded, a process that requires ATP hydrolysis for most proteins. Selectivity of the degradation mechanism is ensured by SP recognition through extrinsic degradation tags such as the *E. coli* SsrA (sequence AANDENYALAA) [19], which are covalently attached at the N- or C-terminus, or intrinsic sequence motifs [20–22]. Flexible, diaphragm-forming loops within the narrow central pore of the unfoldases effect SP unfolding and translocation [23]. These loops, which contain a highly conserved G-aromatic-hydrophobic-G motif [9, 24], are suggested to exert mechanical force on the SP through a “paddling” mechanism [25]. Sequential ATP hydrolysis events within the Clp ATPase ring induce large scale conformational changes of individual subunits [9] that elicit ≃10 Å excursions of pore loops along the ring axis. Strong interaction between the pore loops and the SP coupled with the axial displacement of the pore loops results in application of mechanical force onto the SP.

Biophysical and biochemical experiments have shown that local mechanical stability near the tagged terminus of the SP is correlated with the ATP consumption and degradation rates by the Clp machinery [26]. Destabilization of the highly stable C-terminal *β*-strand of the I27 domain of titin [27] results in greater degradation rates by ClpXP [28] and alteration of the tagged terminus of dihydrofolate reductase, from *β*-strand to *α*-helix or unstructured loop, yielded faster degradation by ClpXP and ClpAP [26]. Increased stabilization of circular permutants of the Green Fluorescent Protein (GFP) resulted in stalling for the variant with a stable intermediate [29, 30].

Recent single-molecule experiments of ClpXP- and ClpAP-mediated unfolding and translocation of multidomain proteins [31–36] used laser optical trapping approaches to restrict the application of force to the N-C direction. The force generation by each central pore loop is reported to be ∼20 pN, corresponding to mechanical work ∼5kT. Discrete steps in unfolding and translocation indicate a power stroke mechanism [32–34]. Studies of ClpXP-mediated threading of GFP in this one-dimensional geometry identified two unfolding intermediates [33, 34, 37, 38]. Competition between refolding and translocation of the first intermediate results in a kinetic constraint in this process [34, 39]. Experiments of Cordova *et al.* [35] and Olivares *et al.* [36] examined multidomain substrates comprising multiple copies of wild-type or V13P and V15P variants of I27 and an N-terminal HaloTag domain. The I27-based construct yielded observation of successive unfolding events of a homogeneous species separated by preunfolding dwell times that reflect the mechanical stability of each domain. The distinct topology of terminal regions of the HaloTag (*α*-helical) and of I27 (*β*-sheet) resulted in distinguishable terminal unfolding events. The opposing force was found to have topology-dependent effect by destabilizing the I27 domains, but decreasing the ClpXP activity in the case of the HaloTag [35]. These results were found to be consistent with the smaller distance to the transition state for the HaloTag than for I27 domains [27].

**Fig 1:**
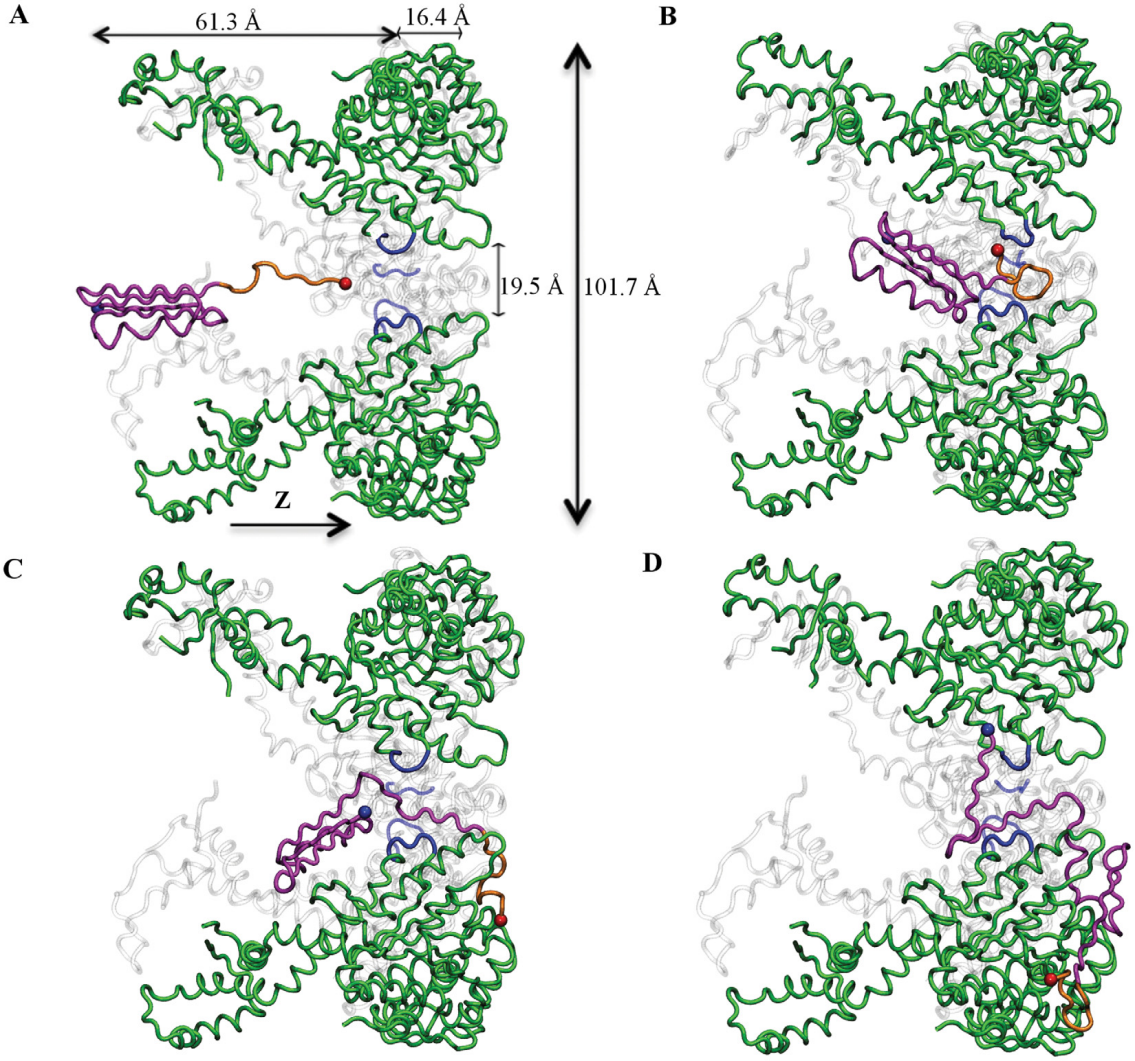
Unfolding and translocation pathway of the *α*/*β* protein by allosteric ClpY. (A) The *α*/*β* model protein (magenta) fused at the C-terminus with the SsrA degradation tag (yellow) binds to the central channel loops (blue) of ClpY (green). The dimensions of ClpY and pore size are indicated. For clarity, two ClpY subunits are not shown and two are shown in gray. (B) The central channel loops engage the substrate protein. Iterative pulling results in unfolding of the substrate to *Q*_*N*_ ≃ 0.7. (C) Following several allosteric cycles, in the absence of the ClpQ peptidase co-factor, the substrate is partially translocated (*Q*_*N*_ ≃ 0.5). (D) The harmonic restraints in the distal region ($z-{\bar{z}}_{loops}>14.5Å$) are added to account for the ClpQ assistance. The allosteric motions of ClpYQ result in complete translocation and refolding of the tagged substrate protein. Molecular images in this paper are created using Visual Molecular Dynamics [58].

**Fig 2:**
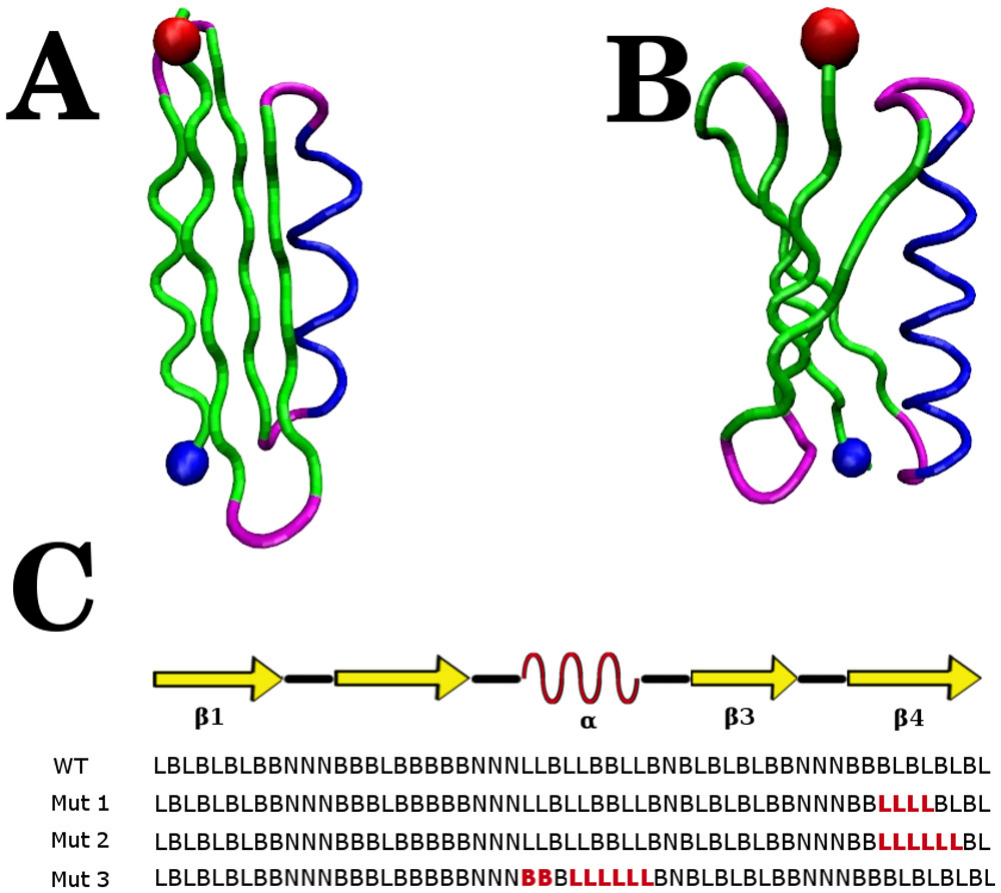
Structural details of *α*/*β* proteins. (A.) Model protein utilized in this study. (B.) Protein L. The *β*-strands are shown in green, *α*-helix in blue, and loop regions in magenta. The C-terminus is indicated by a red sphere and the N-terminus by a blue sphere. (C.) BLN-model sequence (see Methods) of the *α*/*β* model SP and variants with contiguous stretches of non-attractive amino acids (red).

To obtain further insight into these mechanisms, several computational studies of protein unfolding and translocation used coarse-grained approaches that involved mechanical pulling through model pores [40–43] or atomistic descriptions of a non-allosteric ATPase [44, 45]. Details of the allosteric mechanism of AAA+ machines were considered in coarse-grained models of translocation along a biomolecular track [46, 47] and pore opening and closing [48], as well as in the atomistic description of the elementary translocation step [49]. Our group developed coarse-grained models of allosteric cycles of two AAA+ nanomachines, ClpY and p97 [50, 51], that probed complete unfolding and translocation of an all-*α* SP and revealed complex degradation pathways. The coarse-grained description of the system is particularly well-suited for these studies as it enables extensive sampling of the large biological time scales and length scales involved and it provides access to forces and pulling speeds that approach the high end of experimental values. Simulations of mechanical pulling using coarse-grained approaches yield the relative mechanical stability and unfolding pathways of topologically diverse substrates, such as the *β*-barrel GFP [52], the *α*/*β* domain B1 of streptococcal protein L, the all-*α* spectrin [41], the *α*/*β* domain B1 of streptococcal protein G [53, 54] and the *β*-sandwich scaffoldin [55] in very good agreement with single-molecule atomic force microscopy (AFM) experiments. In addition, unfolding pathways are consistent with those obtained from implicit solvent atomistic simulations [41]. The basic premise of the coarse-graining approach, that protein folding mechanisms are guided by contacts that characterize the native structure, has been confirmed by the recent comparison with long atomistic simulations of multiple proteins [56]. Inclusion of non-native interactions in addition to the native contacts, as in the BLN-type model of an *α*/*β* protein (see Methods) developed by Sorenson and Head-Gordon [57], allows us to adequately probe unfolded conformations. In this paper, we use coarse-grained Langevin dynamics simulations to probe ClpY-assisted unfolding and translocation of the *α*/*β* SP (Fig 1) which has the same fold as B1 domains of proteins L and G (Fig 2). This model indicates that unfolding represents the rate-limiting step in the degradation of the *α*/*β* SP. Multiple conformational pathways arise from application of ClpY-induced force along directions of distinct mechanical resistance near the C-terminus of the SP and involve unfolding prior to or simultaneous with translocation. We contrast these results with our previous studies of an all-*α* SP [50] that indicated translocation as the rate limiting step in the degradation pathway. Rapid unfolding of tagged C-terminus of the four helix bundle SP resulted in an obligatory unfolding intermediate three helix bundle. This structure was competent for translocation, however, pathways that included further unfolding were also identified. Taken together, experimental and computational studies reveal strong topology-dependent mechanisms of unfolding and translocation mediated by Clp ATPase nanomachines.

## Results and Discussion

### The native structure of the *α*/*β* protein is not perturbed by the interaction with the non-allosteric ClpY pore

In our simulations, we set the temperature to *T* = 0.7 *T*_*f*_, such that the native secondary and tertiary structures of the isolated *α*/*β* protein are not subject to strong thermal fluctuations [57]. Bulk simulations of the *α*/*β* and *α*/*β*-SsrA proteins (Table 1) confirm the high stability of the native structure at this temperature, as indicated by the large fraction of native contacts, *Q*_*N*_ (S1 Fig). Next, we performed simulations (Table 1) of the native *α*/*β*-SsrA fusion protein and the non-allosteric ClpY in an open pore conformation. To achieve a statistically meaningful number of binding events, we initiate these simulations from configurations in which the SP is located at a minimum distance *d* = 8 Å from the ClpY pore (see Methods). We find that binding of the SsrA tag to the central channel loops of ClpY, which takes place in 359 (≃9% of total) trajectories (Table 1), does not alter significantly the native *α*/*β* structure (S1 Fig). Overall, we surmise that, at this temperature, the native *α*/*β* structure is very robust against interactions with the non-allosteric ClpY pore.

**Table 1:** Summary of Langevin dynamics simulations.

**System**	**SP Sequence**	*v*(*μm*/*s*)^a^	*k*(kcal/(mol⋅Å^2^))	t (*τ*)	N_*traj*_	N_*events*_ ^b^
Bulk *α*/*β*	wild-type	-	-	3.3	112	1
Bulk *α*/*β* − *SsrA*	wild-type	-	-	3.3	112	0
Bulk *α*/*β* − *SsrA*	wild-type	-	-	3.3	48^c^	0
AFM-type pulling	wild-type	8.8×10^4^	3.5	333.3	112	112
ClpY binding	wild-type	-	0	0.167	3864	359
Allosteric ClpY	wild-type	8.8×10^4^	0	50	320	28
Allosteric ClpY	wild-type	8.8×10^4^	0	50	54^c^	12
Allosteric ClpY	wild-type	8.8×10^4^	0.5	70	56	43
Allosteric ClpY	Mut1	8.8×10^4^	0	50	320	22
Allosteric ClpY	Mut2	8.8×10^4^	0	50	320	13
Allosteric ClpY	Mut3	8.8×10^4^	0.5	50	112	77
Allosteric ClpYΔI	wild-type	8.8×10^4^	0	50	56	8
Non-allosteric ClpY	wild-type	-	0.5	35	28	0
Non-allosteric ClpY	wild-type	-^d^	-	3.3	112	112
Non-allosteric ClpY	wild-type	8.8×10^4^	-	3.3	50	37
Non-allosteric ClpY	wild-type	5×10^5^	-	3.3	56	28

^*a*^Effective speed of ClpY active loop motion or constant speed in AFM.

^*b*^The number of successful events observed during each set of simulations. Bulk α/β, Bulk α/β-SsrA and AFM-type pulling: unfolding; Binding: α/β-SsrA binding to ClpY central channel loops; Allosteric ClpY pore, *k* = 0 kcal/(mol·Å^2^): partial α/β-SsrA translocation; Allosteric ClpY pore, *k* = 0.5 kcal/(mol·Å^2^), and non-allosteric ClpY pore: complete α/β-SsrA translocation.

^*c*^These simulations are performed at the temperature *T* = 0.9 *T* *_f_*, while all other simulations are performed at *T* = 0.7 *T* *_f_*. For this protein model, *T* *_f_*≃260K.

^*d*^Pulling at a constant force of 125 pN.

### Atomic force microscopy-like pulling of the *α*/*β* protein results in multiple unfolding pathways that involve sequential or concerted unfolding of hairpins

To examine the bulk mechanical strength of the *α*/*β* protein, we performed AFM-type unfolding simulations by holding the N-terminus fixed and pulling, with constant velocity, the C-terminus along the direction of the termini (Table 1). Unfolding pathways can be discriminated by the ordering of hairpin unfolding events, which are identified by the loss of 50% of inter-strand contacts. We find that the most populated pathway, which occurs in 45% of trajectories, involves the initial unfolding of the N-terminus hairpin. The secondary pathway, found in 41% of trajectories, involves the initial unraveling of the C-terminus hairpin. The remaining 14% of trajectories unfold through a pathway that involves concerted (with resolution 0.15 *τ*) unraveling of both hairpins. AFM unfolding of the model SP occurs very rapidly, with a characteristic first passage time of 0.7*τ* for unfolding at either the N-terminal hairpin or the C-terminal hairpin and forces associated with these unfolding events are in the range of 100—150 pN (S2 Fig). To glean specific features of unfolding of this SP relative to proteins having similar fold, we compare our simulations to results of experimental [59] and computational [60, 61] AFM studies of protein L. Both implicit solvent [60] and $\text{G}\bar{o}$[61] model simulations of protein L identify a single unfolding pathway that involves the shearing of the interface between the C- and N-terminal strands, followed by unfolding of both hairpins. By contrast, for the *α*/*β* protein, the pathway that involves concerted unfolding of the hairpins consists in the simultaneous destruction of the interface between the N- and C-terminal strands and unfolding of individual hairpins, followed by the loss of contacts formed by the two interior *β*-strands and the helix. Although the model protein and protein L have distinct unfolding pathways, the forces required for unfolding the model protein in our coarse-grained simulations are on the same order of magnitude as those determined experimentally for protein L [59]. We attribute the differences in unfolding pathways to the distinct wiring of the two proteins (Fig 2). The model protein is tightly packed, with 118 inter-hairpin contacts and 50 contacts formed between the helix and the two internal *β*-strands, *β*2 and *β*3. By contrast, examination of the crystal structure of protein L reveals that the two hairpins are assembled into a nearly flat *β*-sheet structure with only 24 C_*α*_-C_*α*_ inter-hairpin contacts, established exclusively between the N- and C-terminus *β*-strands, *β*1 and *β*4. We also note that only the C-terminus hairpin forms extensive contacts, 33, with the helix. We surmise that the more complex mechanical unfolding mechanisms of the *α*/*β* protein are due to the tight interfaces involving all secondary structure elements. This conclusion is consistent with results of coarse-grained folding simulations which identify multiple folding pathways for the *α*/*β* protein compared with the single folding pathway of protein L [62].

### Initial unfolding of the SP by ATP-driven ClpY involves the disruption of the C-terminus *β*-hairpin

**Fig 3:**
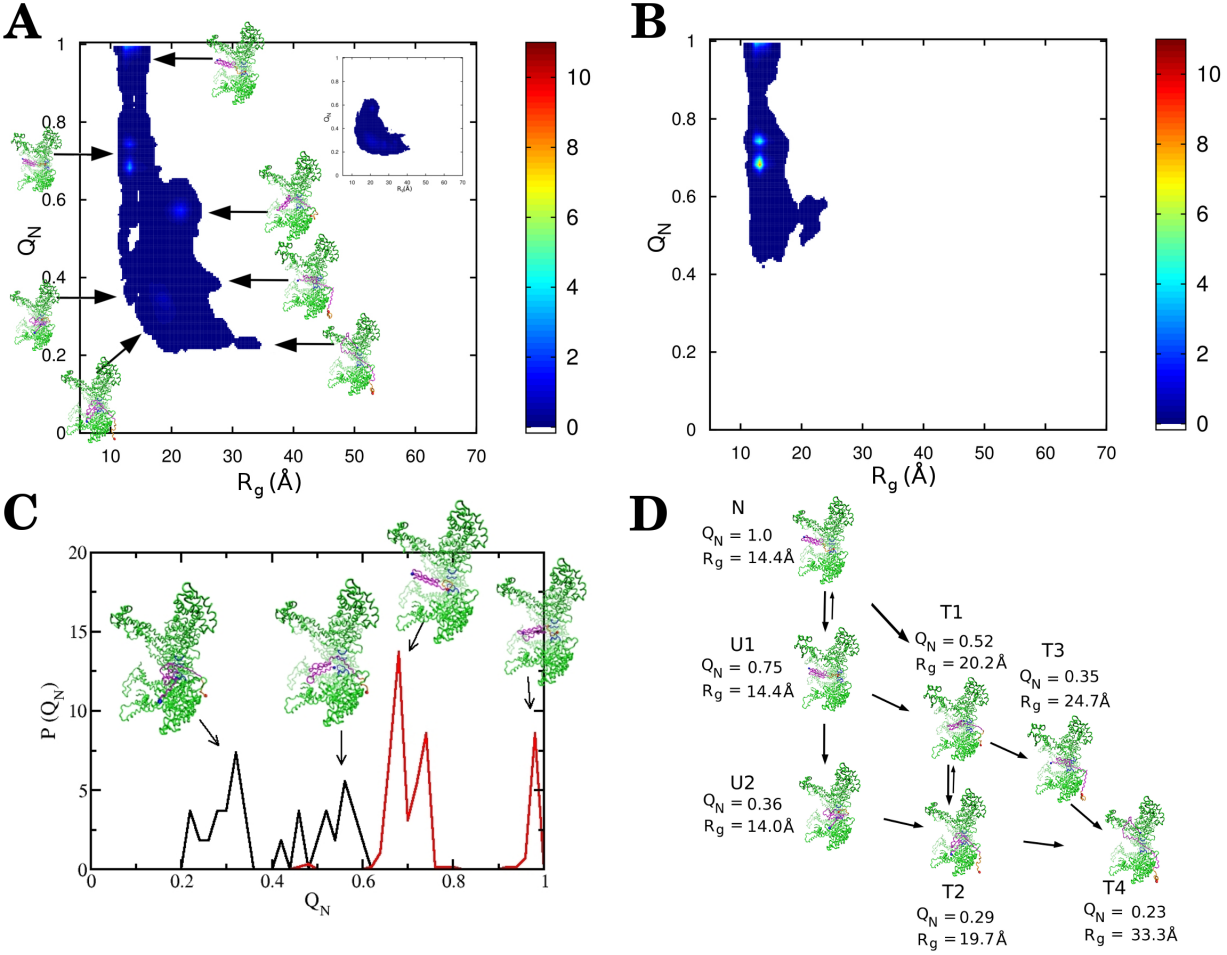
Unfoldase and translocase activity of allosteric ClpY. (A) Probability density maps of the fraction of native contacts *Q*_*N*_ and the radius of gyration *R*_*g*_ for trajectories which result in translocation without assistance from ClpQ. (Inset) Probability density map of the ClpYQ combined action. (B) Map for ClpY trajectories which do not result in translocation. (C) Probability density distribution of *Q*_*N*_ after 50 *τ* for trajectories which result in translocation (black) and trajectories which do not result in translocation (red). (D) Reaction kinetics for unfolding and translocation.

Our computational model [50] describes the ClpY cycle through sequential allosteric motions of pairs of adjacent subunits between their open and closed pore conformations (see Methods). In this model, central channel loops of ClpY have high affinity for the SP during ATP-driven conformational transitions of individual subunits and low affinity otherwise. During the initial ClpY cycles, following binding of the *α*/*β*-SsrA SP to the central channel loops, the SsrA peptide tag experiences intermittent mechanical forces which result in frequently bringing the *α*/*β* protein near the ClpY pore entrance. We find that, within the *t* = 50 *τ* timeframe examined in our simulations (Table 1), SP unfolding (Fig 1(B)) occurs in 82% of trajectories, which is accessed on a time scale of ≃6.5 *τ*. The absence of unfolding events in a subset of trajectories is attributed to the high structural stability of the *β*-strands at the C-terminus. As shown in Fig 3, unraveling of the native structure of the *α*/*β* protein is initiated either by shearing the C-terminus *β*4 strand, which yields the U1 conformation, or by unzipping the C-terminus hairpin simultaneously with translocating the *β*4 strand to establish the T1 state. The U1 conformation is characterized by *Q*_*N*_ ≃ 0.65 − 0.75 and *R*_*g*_ ≃ 14 Å and it has a root mean square deviation (RMSD) of ≃ 2.1 Å with respect to the native structure. The reversible unfolding event that leads to the U1 conformation primarily disrupts contacts formed by the *β*4 strand with the protein core and, as a result, the U1 conformation retains a globular shape that precludes its translocation. Additional unfolding prior to translocation (Fig 3), which yields the U2 conformation, consists of unraveling and removing the C-terminal *β*4 strand from the remaining intact structure. Both U1 and U2 conformations are compatible with translocation, which yields conformations that retain high (T1) or low (T2) native content. Overall, as illustrated in Fig 3(D), we identify both “direct” pathways, in which the work performed by the Clp ATPase results in simultaneous unfolding and translocation, and “indirect” pathways that involve SP unfolding (U1 or U2) prior to initial translocation (Fig 1(C)) or refolding due to exchanges between the T1 and T2 states. After 50 *τ*, trajectories which result in translocation populate more unfolded states than those which do not result in translocation (Fig 3(B) and 3(C)). This unfolding mechanism initiated by unraveling from the C-terminus is in accord with experimental studies [26] and our previous simulations of HBP translocation by ClpY [50].

### Translocation occurs in cooperative bursts of secondary structural elements

The rate of translocation obtained in our simulations is much lower than that of unfolding, with only 9% of the simulation trajectories resulting in SP translocation (Table 1 and Fig 1(D)). This low translocation rate reflects the limited ability of ClpY to unravel a sufficiently long segment at the C-terminus of the SP in the initial unfolding event. Consistent with this, initial translocation, which occurs on the direct pathway with mean first passage time of ≃7 *τ*, includes the SsrA tag and an average of five amino acids at the C-terminus of the *α*/*β* protein. The indirect pathway that includes the N → U1 → T1 transitions takes place on a slower time scale with first passage time of ≃10 *τ*. Overall, during the 50 *τ* duration of ClpY simulations, we find that the high stability of the C-terminal region of the *α*/*β* SP results in translocation of only seven of the ten amino acids of the *β*4 strand. We also probe temperature-dependent effects on the translocase function of ClpY, as degradation of heat-denatured proteins is an important function of proteases. To this end, we performed additional simulations at *T* = 0.9 *T*_*f*_ (Table 1). While the native conformation of the bulk SP is largely preserved at this higher temperature, it is more easily destabilized by interaction with the allosteric ClpY and unfolding events occur in all of the simulation trajectories. Translocase activity is enhanced significantly at higher temperature, with 26% of simulation trajectories resulting in SP translocation. Nevertheless, the average contour length of the segment translocated, 31.8±23.7 Å is similar to that at lower temperature, 26.6±27.7 Å, due to long time scales associated with individual translocation steps. We note that competing events of SP binding to the auxiliary I domain of ClpY, reverse translocation and refolding play an increasing role at higher temperature and preclude observation of complete SP translocation events during the 50 *τ* simulations performed. We surmise that efficient protein degradation under heat stress is mediated by fast initiation of SP translocation and processing by the peptidase.

**Fig 4:**
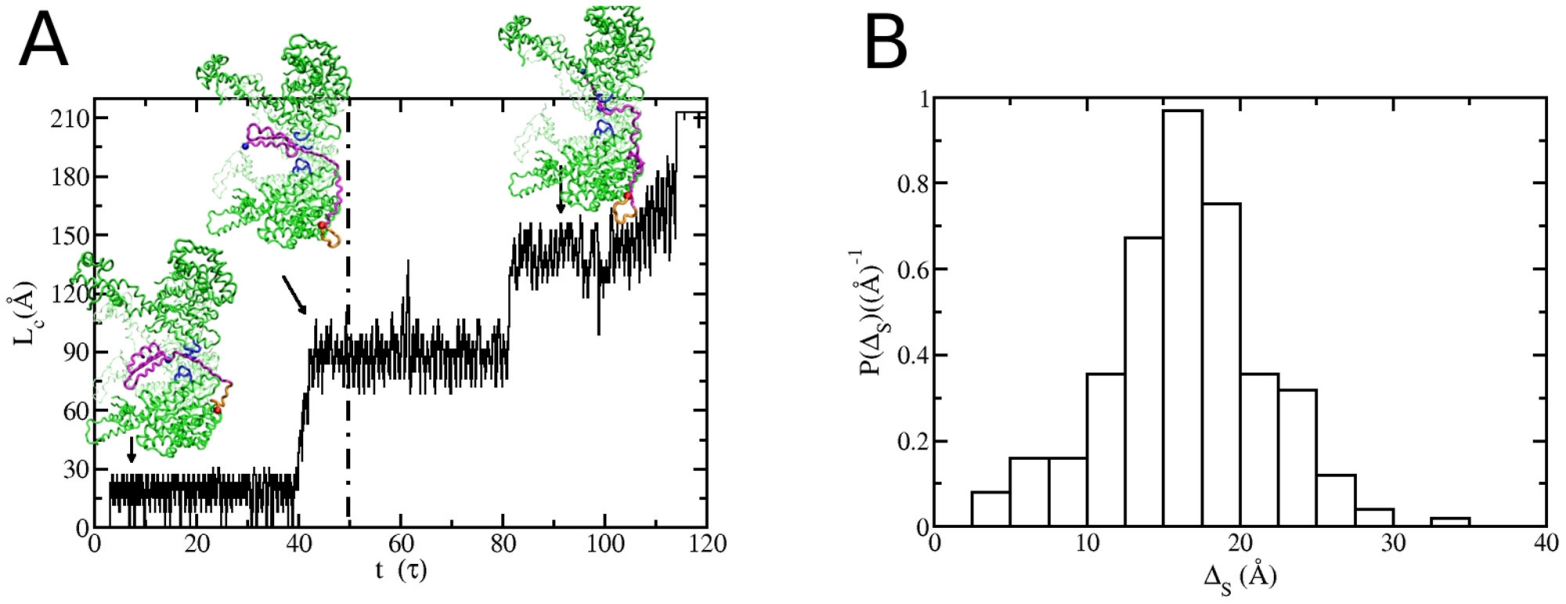
Translocase action of the allosteric ClpY pore. (A) Contour length of the polypeptide translocated through the pore as a function of time. After 50 *τ* harmonic restraints are applied in the distal region to mimic the interaction between the SP and ClpQ. (B) Probability density distribution of the end-to-end extension of the translocated segment.

Association of the ClpY nanomachine with the peptidase compartment ClpQ enhances SP translocation efficacy by a factor of two to three. To account for the peptidase contribution to translocation we apply a weak harmonic restraint to SP amino acids that have been translocated to the distal region of ClpY (see Methods). The small value of the force constant, *k* = 0.5 kcal/(mol⋅Å^2^), ensures that SP translocation is driven by ClpY allostery. Simulations that mimic the ClpYQ action (Table 1) are initiated from the partially translocated conformations that result from the independent ClpY action. To confirm that allosteric motions are responsible for the dominant contribution to SP translocation, we performed control simulations involving non-allosteric ClpY pores (Table 1). Our results, shown in S3 Fig, indicate that, in the absence of ClpY allostery, an SP segment with average contour length of 38.5±14.2 Å is translocated compared with allosteric-driven translocation of a segment with average length of 115.1±10.4 Å. As shown in Fig 4(A), combined action of allosteric-driven motions of ClpY and assistance from ClpQ result in complete SP unfolding and translocation. In the single translocation trajectory illustrated in this Fig, the C-terminus *β*-hairpin is translocated through the unassisted ClpY action, which corresponds to a segment with contour length ≃ 90 Å. After the distal restraint is applied, multiple cooperative transitions take place which yield translocation with contour length of ≃ 50–75 Å. In these simulations, the first passage time for complete translocation of the *α*/*β*-SsrA is ≃32 *τ*. Using the value of contour lengths associated with each transition, we estimate the distribution of end-to-end extensions (Δ_*S*_) of translocated segments of a polypeptide chain with persistence length 6.5 Å. As shown in Fig 4(B), the maximal translocation step involves Δ_*S*_ ≃ 30 Å and the average extension of the translocated segment is 〈Δ_*S*_〉≃20 Å. Translocation transitions occur within a single cycle (Δ*t* ≲ 1 *τ*) and the average pause time between transitions, is ∼7 *τ*. These results are in accord with single molecule studies of ClpX-mediated translocation [32–35], which indicate that translocation of polypeptide segments involves individual steps of 1, 2, 3, or 4 times the *l* ≃ 10 Å axial excursion of a single ClpY loop. The length of translocation steps commensurate with loop excursions is attributed to single or multiple power strokes. In addition, coordinated translocation events within a single ATPase cycle indicate collaboration between several Clp subunits to promote translocation.

As a model for intra-ring communication, Cordova *et al.* [35] proposed that stochastic firing of one ClpX subunit triggers a coordinated chain of ATP hydrolysis or release events in the remaining subunits to generate additional power strokes. The total number of such events, which may proceed sequentially or stochastically through interaction with neighboring subunits, is limited by the asymmetric ring loading with up to 4 nucleotides under saturating conditions [63, 64]. In our simulations, the upper bound of the length of translocation steps, Δ_*S*_ ≤ 3 *l*, is consistent with the description of the ClpY allosteric cycle to comprise six two-subunit moves. Thus, between one and three ClpY loop excursions can promote polypeptide translocation within the productive hemicycle. Tight binding between all subunit loops and the SP in the “closed” pore conformation and SP release during the pore opening hemicycle reset the ClpY loops-SP interactions at the end of each ClpY cycle, therefore SP binding to the active loop at the beginning of the next cycle is a stochastic event. We caution that the two-subunit moves described in our model should not be interpreted as concerted actions of subunits as proposed in the case of the archaeal homolog PAN by Smith *et al.* [65]. In our simulations, crystal structures used to describe the “open” and “closed” pore conformations include asymmetric and predetermined nucleotide states of subunits (see Methods), therefore the six ClpY loops have divergent ability to promote translocation. To examine in detail the effects of directionality of allostery on unfolding and translocation, our previous study of ClpY and the double-ring p97 nanomachines [51] included a “6×1” hemicycle description comprising clockwise, counterclockwise or random intra-ring ordering of the six subunit moves. We found that, while each of these allosteric modes results in unfolding and translocation, the clockwise direction is the most efficient due to structural bias in loop motions. During each subunit move, the associated loop imparts clockwise torque onto the SP and therefore effectively biases SP handling in this preferential direction. In addition, we considered ClpY variants with subunit loops that have reduced interaction with the SP or impaired conformational transitions. Our simulations indicated that ClpY variants with two mutant loops have translocase activity similar to the wild-type machinery. We also found that variants with at least three catalytically-active subunits, in partially contiguous configuration, maintain translocase function. These findings are in accord with recent experimental studies of ClpX variants by Iosefson *et al.* [66], which indicated that a subset of the six wild-type loops suffices for efficient degradation of unfolded I27 and folded GFP variants. Further development of our model, in particular incorporating high-resolution structures of distinct asymmetric ClpY intermediates as they become available, will provide enhanced access to detailed loop-SP interactions during the ATPase cycle and could result in greater predictive power for simulations. In particular, this enhanced model would allow us to quantify the effect of the probabilistic [67]*vs.* predetermined sequence of subunit firing events on unfolding and translocation activity.

### Intermittent forces exerted by central channel loops of ClpY effect SP unfolding and translocation

**Fig 5:**
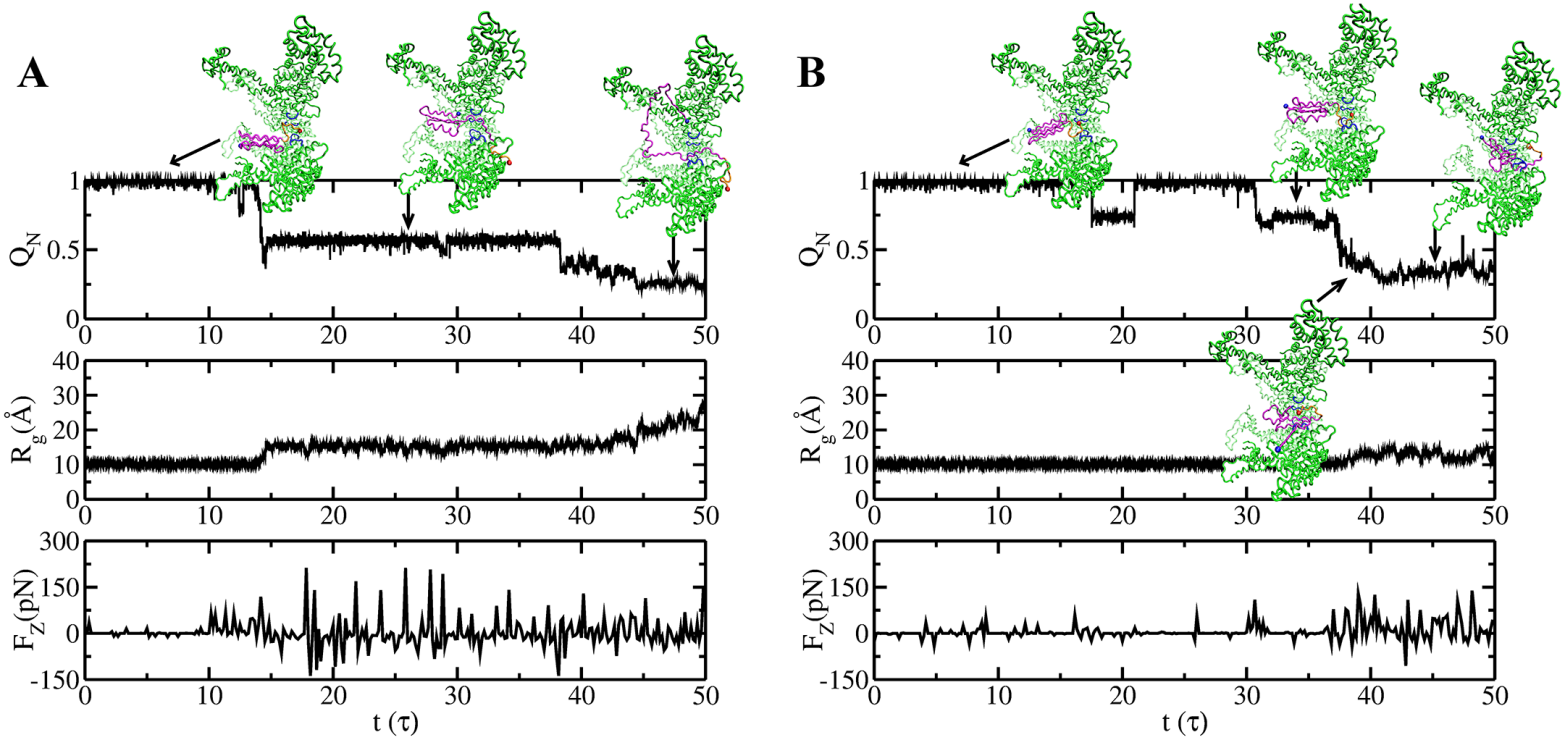
Unfolding and translocation pathways effected by repetitive forces exerted by the ClpY channel loops onto the SP. Time series of the fraction of native contacts, *Q*_*N*_, the radius of gyration *R*_*g*_ of the SP, and forces along the pore axis *F*_*z*_ in individual trajectories that result in (A) simultaneous unfolding and translocation or (B) unfolding prior to translocation. Reversibility of initial unfolding is observed in (B).

To glean the detailed mechanical action effected by the ClpY ATPase, we analyze the time series of forces exerted onto the SP (Fig 5). To this end, we compute the average force exerted by central channel loops of ClpY in each step of the cycle. Fig 5 illustrates the time series of axial forces and their effect on SP unfolding (*Q*_*N*_) and translocation (*R*_*g*_) in trajectories that probe simultaneous or separate events. In both types of pathways, we find that translocation requires axial forces of 75–130 pN. During each trajectory, forces are applied intermittently onto the SP, indicating stochastic events of SP gripping by the ClpY loops. The magnitudes of these intermittent axial forces are distributed in a wide range of values, which supports the power stroke mechanism. As noted above, in trajectories that involve unfolding prior to translocation (Fig 5(B)), the initial unfolding is reversible as a result of the combination of relatively weak forces and their intermittent application.

### Moderate interactions of the SP with the I-domain do not assist in the initial translocation event

The auxiliary I-domain (residues 110—243), which is specific to ClpY, actively assists degradation of the Arc repressor substrate through a proposed mechanism that involves restricting SP mobility on the proximal pore side [68]. In accord with experimental studies, computer simulations of an all-*α* SP indicate that the I domain binds and stabilizes the unfolded SP [50]. The deletion of the I-domain in ClpY variants was shown to drastically reduce the ATPase activity of ClpY [68] and to suppress degradation of specific SPs [10]. In the case of the *α*/*β* SP, we find that interactions with the I-domain occur with energy *E_I_* ≳ −20 kcal/mol (S4 Fig). This interaction energy is significantly weaker than that found for the four helix bundle SP, which involves interaction energies ≳ −100 kcal/mol [50]. The distinct interaction of the two SPs with the I-domain is consistent with their unfolding and translocation mechanisms. The unfolding of the four helix bundle occurs very rapidly (≃1 *τ*) whereas its translocation takes place on a long time scale (≃25 *τ*). Prior to translocation, the unfolded SP, which expose hydrophobic residues, interact moderately with the I-domain. These interactions stabilize the unfolded conformations allowing the pore loops to effectively exert force onto the C-terminus of the SP. By contrast, unfolding represents the rate limiting step in the degradation pathway of the *α*/*β* SP. As noted above, unfolding of the C-terminus of this SP is either simultaneous with the initial translocation event, on a ≃7 *τ* time scale, or it precedes translocation by ≃3 *τ*. The unfolded conformation of the *α*/*β* SP maintains significant native content and it does not require significant external stabilization. As a result, the unfolded intermediate is rapidly translocated or refolding of the SP occurs. These competing events yield weak interaction of the SP with the I-domain even in the absence of translocation. We find that, in 95% of trajectories that do not result in translocation the interaction between the SP and the I-domain is weak, with *E*_*I*_ ≃ 0 kcal/mol (S4(b) Fig). Consistent with these observations, additional simulations (Table 1) indicate that the I-domain deletion mutant of ClpY maintains translocase activity similar to the wild type ClpY. Nevertheless, the initial unfolding event occurs much later than in wild-type simulations, with a mean first passage time of ≃18 *τ*, highlighting the steric effect of the I-domain that restricts the rotational mobility of the SP.

We propose that the effect of the I-domain on the translocase activity of ClpY is dependent on the SP stability and the interplay between unfolding and translocation mechanisms. For SPs with weak stability, unfolding and translocation may occur on significantly different time scales and the initial unfolding event results in exposing a large number of amino acids located in the hydrophobic core of the SP. The I-domain helps to stabilize this unfolded conformation and assists translocation by reducing the conformational flexibility of the SP. By contrast, if the native character of the SP is preserved during the timeframe between unfolding and translocation, the I-domain interacts weakly with the unfolding intermediate. This hypothesis is consistent with the selective effect of I-domain deletion observed in experimental studies [10, 68] and with results of computational studies of model proteins [50].

### Translocase activity tolerates polypeptide tracks that are not gripped by the ClpY loops

Following the initial step of threading the degradation tag through the Clp ATPase pore, complete SP translocation requires sustained pulling of the polypeptide chain. Given the Clp ATPase versatility in processing proteins with diverse sequence, it is important to understand the effect on translocation of weak interactions between regions of the unfolded SP chain and Clp loops. To this end, we performed substitutions within the *α*/*β* sequence that yield variants with distinct length and location of loosely gripped regions (Fig 2(C)). In our model, these regions consist of contiguous stretches of four or six hydrophilic amino acids with the contour length of the hydrophilic stretch, Δ_*L*_ ≥ *l* = 10 Å. The Mut1 variant has Δ_*L*_ ≃ 10.5 Å, while variants Mut2 and Mut3 have Δ_*L*_ ≃ 17.5 Å. The location of amino acid substitutions is chosen near the C terminus (Mut1 and Mut2) or at internal sites (Mut3) of the *α*/*β* substrate (Fig 2(C)). To efficiently probe the effect of these mutations on translocation we use a fast-forwarding approach. For each *α*/*β* variant, simulations are initiated from configurations that involve SP intermediates with mutated segments located in unfolded regions that are in contact with the ClpY pore. For C-terminus variants Mut1 and Mut2, the initial configuration corresponds to the unfolded and not translocated state U1 (Fig 3(D)). For the internal Mut3 variant, simulations are initiated in the unfolded and partially translocated state U3 (Fig 3(D)). As shown in Table 1, all of the SP variants considered are viable for translocation albeit at reduced rates compared to the wild-type SP, indicating that “slippage” of the ClpY loops over stretches with contour length exceeding a single loop excursion (Δ_*L*_ > *l*) of the SP is tolerated. We find that, in the more stringent cases considered, 4% of the trajectories involving Mut2 result in translocation compared with 9% for the wild-type SP, whereas 69% of Mut3 trajectories result in translocation *vs*. 77% in the wild-type SP.

**Fig 6:**
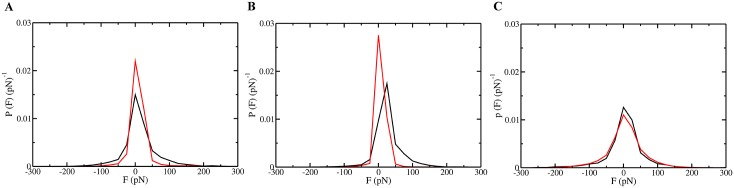
Forces exerted by the ClpY central channel loops onto SP variants. Probability density distributions of the axial force applied by active ClpY loops onto the *α*/*β* SP (black) and its variants (red) while the mutated or corresponding wild-type polypeptide segment is located within the pore. (A) Mut1, (B) Mut2, and (C) Mut3. Curves corresponding to wild-type distributions differ due to the distinct length and sequence of the segments targeted in each mutation.

The length and location-dependent effect of mutations on translocation is consistent with the distribution of forces applied by the ClpY loops onto variant and wild-type SP regions (Fig 6). Mut1 and Mut2 variants experience a reduced grip within the ClpY pore compared with the wild-type case (Fig 6(A) and 6(B)). In the wild-type case, forces larger than 50 pN represent a distinctive tail of the distribution and provide a frequent opportunity for translocation. By contrast, forces applied onto Mut1 and Mut2 variants are weaker and they are dominated by the SP slippage events (*F* ≃ 0 pN). The relatively small effect on translocation of mutations at internal sites (Mut3) is consistent with the similar distribution of forces applied to internal regions. In this case, weak slippage of the mutant SP chain is due to the additional pulling assistance from the peptidase which is modeled by harmonic restraints in the distal region.

**Fig 7:**
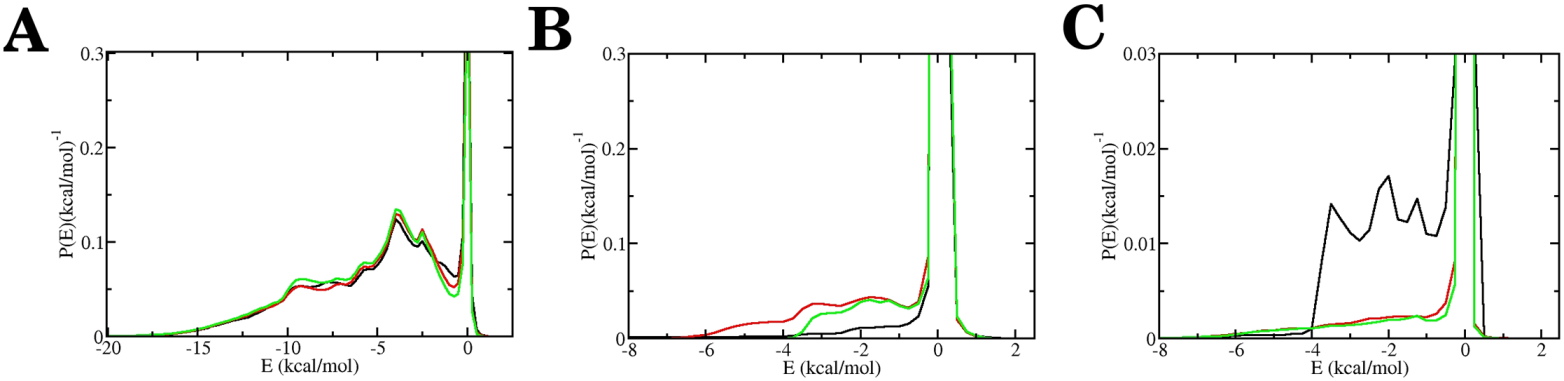
Interactions of SP variants with ClpY central channel loops. Probability density distribution of the interaction energy *E* between the central central loops and (A) the SsrA degradation tag, (B) *β*4 strand and (C) the *β*3 strand. The curves shown represent results obtained for Wild Type (black), Mut1 (red) and Mut2 (green) sequences.

To ascertain the microscopic interactions that underlie the translocation behavior of the SP variants Mut1 and Mut2, we determine the interaction energy between the secondary structural elements near the mutated region and the active loops of ClpY. Fig 7 shows that the energy distributions of interactions involving the SsrA tag, *β*4 and *β*3 strands record the relative ability of the ClpY loops to promote translocation of the SPs. The interaction of the SsrA tag region with the ClpY loops is nearly the same in all three SP variants (Fig 7(A)) as the tag is not affected by the mutation. By contrast, the strong grip of the ClpY loops on the wild-type SP *β*4 strand results in the rapid displacement of this region and relatively less frequent sampling of strong interaction events than for the corresponding region in the Mut1 and Mut2 variants (Fig 7(B)). Interestingly, the Mut1 variant represents a balancing of strong interactions that act to promote translocation, and slippage events that result in the *β*4 strand being localized frequently within the ClpY pore. The Mut2 variant disfavors the interactions between the ClpY loops and the *β*4 strand, reducing the sampling of the large energies and the likelihood of translocation. The interaction of the *β*3 strand with the ClpY loops is weak in both the Mut1 and Mut2 variants due to the infrequent translocation events that prevent localization of this region within the ClpY pore (Fig 7(C)). In the wild-type case, frequent sampling of large energy events is noted due to the convergence of two factors. Greater translocase activity promotes this strand within the pore, while the unassisted translocation capacity is reached and it prevents advancement of the *β*3 strand into the distal region.

Our results, which support translocation of SP variants with slippery tracks of 4–6 residues, are consistent with experiments indicating that the length of Glycine-Alanine repeats inserted in an SP construct controls the inhibitory effect of ClpXP degradation [69]. Repeats consisting of 7 or 8 residues result in production of ≲ 50% intermediates, whereas repeats of 9, 10 or 15 residues yield >60%. Longer regions of low complexity sequences stall proteasomal degradation altogether, as shown by attaching a 37-residue Glycine-rich region to dihydrofolate reductase SP [70] or a 30-residue Glycine-Alanine repeat to the mouse ornithine decarboxylase [71]. Reduced grip of the proteasome or Clp ATPase on the SP is functionally exploited *in vivo* to partially process proteins. As an example, the proteasome regulates the activity of the NF-*κ*B transcription factor, which contains a Glycine-rich region, to yield the p50 fragment from the p105 precursor [70]. In *Caulobacter crescentus*, ClpXP generates the shorter *γ* form of the ATP-binding clamp loader subunit DnaX from the longer *τ* form by stalling the proteolytic process through a Gly-rich tract [72].

### Mechanical pulling through a non-allosteric pore yields pathways that involve simultaneous SP unfolding and translocation

In previous work [50], we found that allosteric-driven motions of the ClpY ring result in distinct unfolding and translocation pathways of an all-*α* SP from those identified by mechanical pulling of the SP through the non-allosteric ClpY pore. To glean the effect of SP topology on these mechanisms, we perform mechanical pulling, using either a constant force or a constant velocity approach, of the *α*/*β* substrate through a non-allosteric pore in the “open” (ATP-bound) conformation (see Methods).

**Fig 8:**
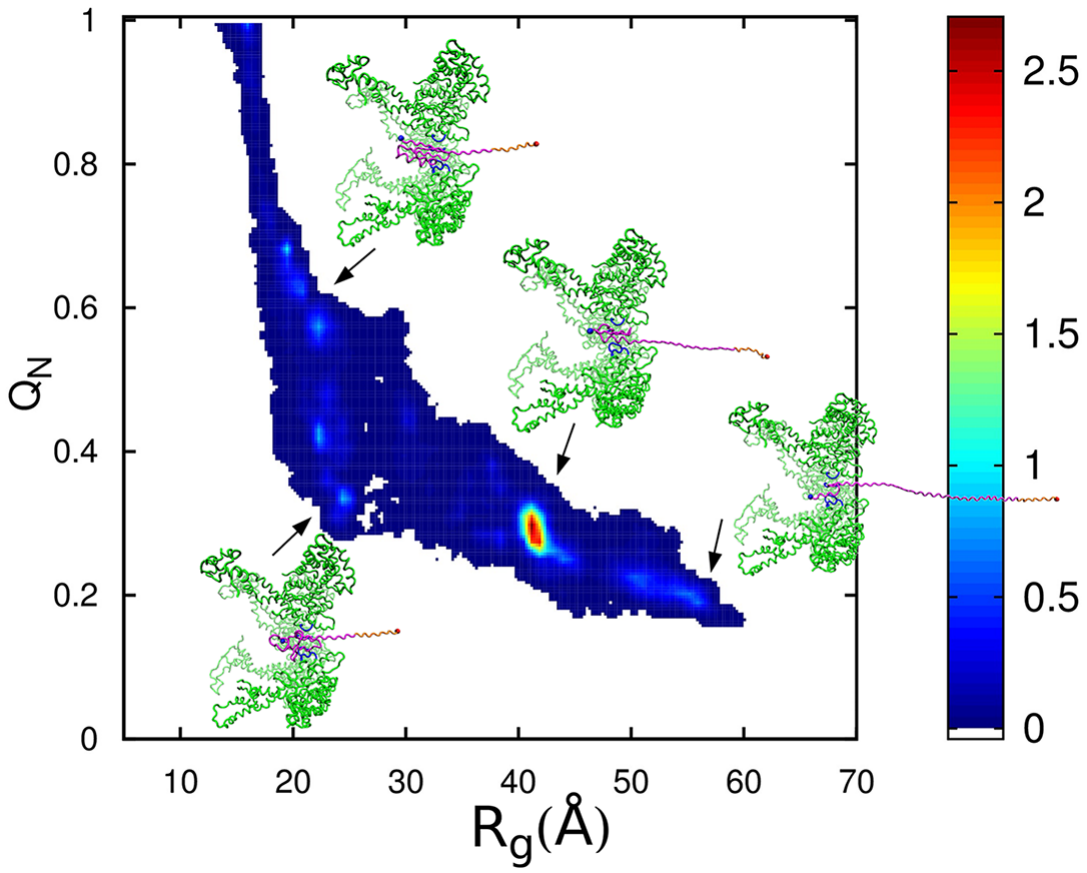
Unfolding and translocation by mechanical pulling with a constant force through a non-allosteric ClpY pore. Probability density map of the fraction of native contacts and radius of gyration for simulations of mechanical pulling through a non-allosteric pore with constant force *F* = 125 pN.

In the constant force approach, the SP is pulled with a force of 125 pN, which corresponds to the threshold value required to unfold and translocate the SP. In all trajectories, the unfolding of the SP is initiated at the C-terminus and translocation takes place concurrently with unfolding. The major unfolding pathway, which is identified in ≃ 68% of trajectories, involves complete SP unraveling by sequential removal of secondary structural elements (*β*4, *β*3, *α*, *β*2, *β*1) from the SP core. The remaining trajectories involve translocation of partially folded structures comprising the intact hairpin 1 (*β*1*β*2) or hairpin 1 and the *α*-helix (*β*1*β*2*α*). As shown in Fig 8, these multiple translocation pathways result in a broad range (0.2 ≤ *Q*_*N*_ ≤ 1) of unfolded SP conformations sampled that overlaps with the corresponding range in the allosteric-driven mechanisms. Nevertheless, continuous application of force yields poor sampling of unfolded and not translocated SP conformations (0.2 ≤ *Q*_*N*_ ≤ 0.8 and 10 Å ≤*R*_*g*_≤ 20 Å). Translocation of partially folded structures is favored by the large diameter of the pore and the compact *β*-folded structures, as well as the large pulling force. Although sampling of these partially folded structures is enhanced by these factors, translocation of segments with intact secondary structure is realistic. Experimental studies have shown that disulfide bonded SPs are translocated and degraded by ClpXP [73, 74], a homolog of ClpY. The average time required for complete unfolding and translocation by pulling through a rigid pore with constant force is ≃ 0.6 *τ*, nearly an order of magnitude faster than in the case of allosteric ClpY. While constant force pulling simulations are able to reproduce the initial unfolding and translocation events of the *α*/*β* SP found in simulations of allosteric ClpY (unraveling at the C-terminus followed by translocation of the unfolded polypeptide), the time scales are much faster and additional unfolding and translocation pathways are not in agreement. These results are in accord with findings in our previous simulations of the four helix bundle substrate [50] which indicate faster unfolding and translocation time scales and distinct unfolding pathways in constant force simulations compared to those obtained in allosteric simulations. The studies of the *α*/*β* SP, however, yield multiple pathways of unfolding and translocation in the constant force simulations and result in better sampling of partially translocated conformations.

**Fig 9:**
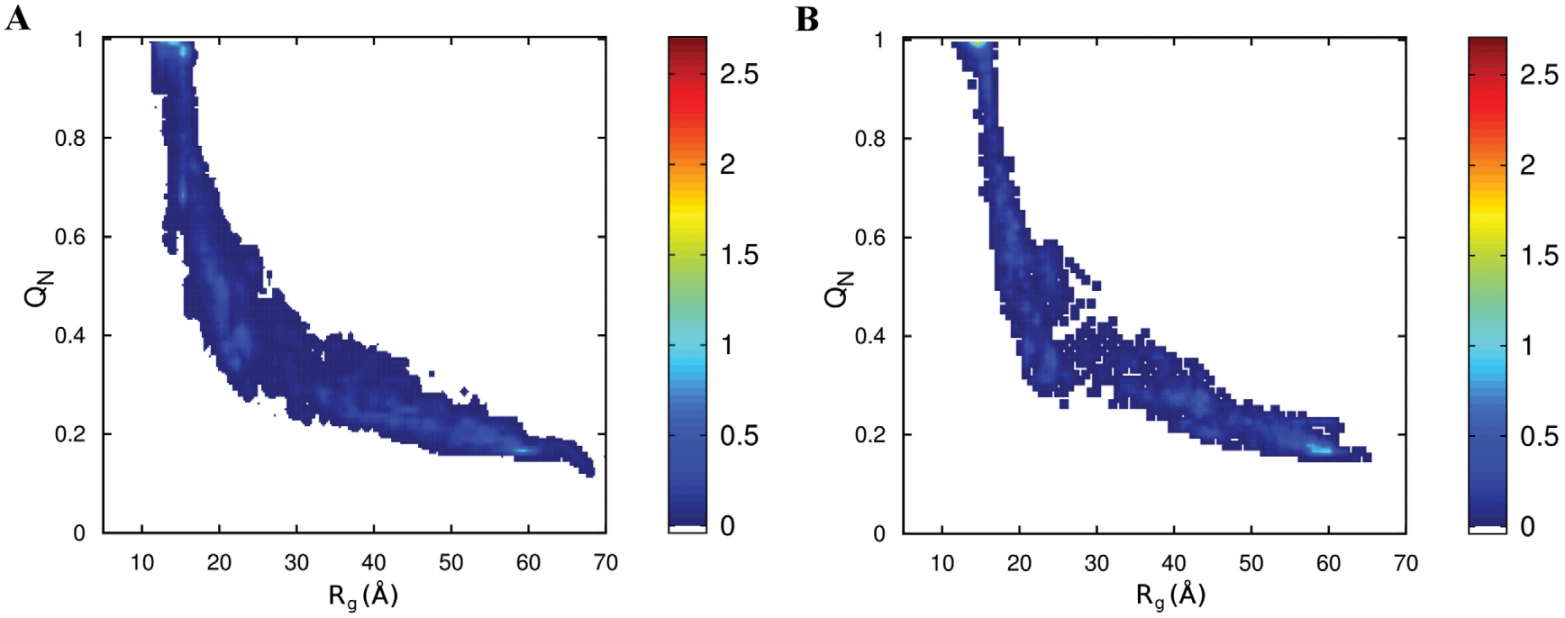
Unfolding and translocation by mechanical pulling with a constant velocity through a non-allosteric ClpY pore. Probability density maps of the fraction of native contacts and radius of gyration for simulations of mechanical pulling through a non-allosteric pore with constant velocity of (A) *v* = 8.8 × 10^4^ *μm*/*s* and (B) *v* = 5 × 10^5^ *μm*/*s*.

In the constant velocity approach, we performed simulations that involve pulling the *α*/*β*-SsrA SP at speeds of 8.8 × 10^4^ *μ*m/s or 5 × 10^5^ *μ*m/s (Table 1). Our simulations result in a single translocation pathway that consists in unraveling the SP at the tagged C-terminus followed by simultaneous translocation of the unfolded structure (Fig 9). The SP interacts favorably with the I-domain and surface surrounding the entrance to the central channel, which facilitates unfolding. These interactions are stronger in this case that in the allosteric case due to the continuous application of external forces. At slower speeds, these off-axis interactions may yield unsuccessful events as the SP is likely to be deflected off the axis of the channel (Table 1). We find that the critical forces necessary for unfolding and translocation in the constant velocity simulations exceed those found in both constant force pulling and allosteric simulations (S5 Fig). Thus, the constant velocity approach highlights the importance of the local stability near the tagged C-terminus of the SP for unfolding and translocation, which is in accord with experimental findings [26]. Nevertheless, the single unfolding and translocation pathway that emerges in this type of simulations provides limited sampling of SP conformations beyond this initial event.

### Conclusions

Clp proteases are versatile nanomachines which are able to degrade substrate proteins with diverse topology provided that a degradation tag is attached for recognition. While experiments revealed the strong correlation of SP topology and degradation rates, molecular details of these processes are insufficiently understood. Using molecular dynamics simulations of a coarse-grained model of ClpY allostery, we investigated the complete unfolding and translocation of an *α*/*β* SP. To glean the dependence of Clp ATPase-assisted processing on topology, we compared and contrasted ClpY-mediated remodeling of this substrate with our prior results of an all-*α*SP.

Our results reveal that for both topologies unfolding is initiated at the tagged C-terminus and translocation takes place in discrete steps. Translocation of polypeptide segments of lengths 10–30 Å, which represent multiples of the 10 Å axial motion of single loop, highlights intra-ring allosteric cooperativity that enables successive SP handling by several subunits. Multi-subunit engagement of SPs during single ATPase cycles enables the machinery to promote translocation of weakly gripped polypeptide chains and to overcome kinetic constraints due to refolding reactions. These aspects are in agreement with single molecule experiments [32–35, 38] that involve mechanical pulling applied by ClpY loops along the C-N direction of SPs. Conservation of these mechanisms for diverse topologies and using unidirectional or multi-directional pulling geometries emphasize the active role of central pore loops in the mechanical action of Clp ATPases.

Topology-specific mechanisms arise primarily from the local mechanical stability of the SP near the tagged and the direction of force application. Unfolding of *α*/*β* SP involves either shearing the C-terminal *β*-strand or by unzipping the C-terminal *β*-hairpin. These distinct unfolding pathways indicate that mechanical forces generated by the central channel loops are applied along multiple directions of the SP. Based on these results, we propose that *the minimal requirement for unfolding mediated by Clp ATPases is for SPs to possess directions of weak mechanical resistance near the tagged terminus.* We also find that the rate-limiting step in the degradation process is strongly dependent on the SP topology. For the weakly bound all-*α* four helix bundle SP [50] translocation is the rate-limiting step, while for the *α*/*β* SP unfolding represents the rate-limiting step. Assistance from auxiliary I-domains of ClpY is also dependent on SP topology leading to a passive role in unfolding and translocation through steric constraints, as in the case of the *α*/*β* SP, or an active role in stabilizing the unfolded conformation and assisting sequential translocation, as in the case of the all-*α* SP. Our simulations yield several predictions testable by experimental studies. Specific mechanisms of unfolding and translocation proposed to result from the directionality of the ClpY-mediated force can be probed in single molecule studies of SP variants with engineered N and C termini or with restricted unfolding pathways. Translocation cooperativity is suggested to be modulated by secondary structure and may be probed in comparative studies of proteins with diverse secondary structure.

Internal structural elements of the SP can also play an important role in translocation, as in the case of disulfide-bonded or knotted proteins, or when the SP recognition by the Clp ATPases involves internal degradation tags [74, 75]. For disulfide-bonded chains and polypeptides engaged at internal sites, translocation of multiple chains has to occur simultaneously which imposes specific kinetic constraints. Interestingly, the wider multi-chain substrate is accommodated without distortion of the ClpX pore [73]. Degradation of knotted proteins is particularly intriguing as mechanical force applied at the protein ends results in knot tightening. Consequently, in mechanical pulling of knotted biopolymers through narrow pores several outcomes are possible depending on the relative size of the knot to the pore width, $R_{g}^{knot}/d_{pore}$, the external force applied and the location of the knot along the chain. As illustrated by computer simulations [76], the intact knot can be translocated through a rigid cylindrical pore albeit at a significantly slower translocation rate and through distinct intermediate conformations of the knotted protein compared with the unknotted chain. At narrow pores, tightened knots are not translocated, but remain pinned to the surface and could jam the pore if the pulling force exceeds a threshold value [77, 78]. Nevertheless, low forces facilitate reptation-type moves of the polymeric chain through the knot, effectively resulting in knot diffusion towards the free polymer end and chain translocation [77]. A similar mechanism has been proposed to allow knotted protein translocation [78]. These results suggest as plausible mechanisms of Clp-mediated translocation of the knotted proteins both the diffusion of the knot along the protein chain through the repetitive action of the pulling force and the internalization of the intact knot through the fluctuating Clp ATPase pore. Further experimental and computational studies are needed to discriminate these possible mechanisms of degradation of knotted proteins by the ClpXP.

Clp-assisted unfolding and translocation mechanisms are distinct from mechanical unfolding by AFM-type simulations, which reveal multiple unfolding pathways from the C-terminus, N-terminus, or simultaneously from both termini. Mechanical unfolding and translocation by pulling through a rigid ClpY pore with constant velocity or constant force are able to reproduce some of the molecular details such unfolding from the C-terminus and the conformations sampled, though larger effective forces are required when pulling with a constant velocity, while pulling with constant force results in simultaneous unfolding and translocation and some unphysical pathways.

## Methods

### Coarse-grained model of the ClpY ATPase-SP interaction

In order to overcome large length scales and to reach biologically relevant time scales associated with Clp ATPase-mediated unfolding and translocation, we developed coarse-grained models of these systems. The ClpY ATPase and the SP are represented by using an “united atom” model that describes each amino acid as a single bead located at the *C*_*α*_ position [79]. Three amino acid types are distinguished, hydrophobic (B), hydrophilic (L), or neutral (N) [80]. We use the CHARMM molecular modeling program [81] to perform coarse-grained Langevin dynamics simulations of these systems. Using this approach, we obtain multiple simulation trajectories (Table 1) that result in complete SP unfolding and translocation during repetitive ATPase cycles.

### Model for the substrate protein (SP)

To probe the effects of ClpY-mediated unfolding and translocation, we use the BLN model of the 56-amino acid *α*/*β* substrate protein developed by Sorenson and Head-Gordon [57]. The Hamiltonian of this model is *H* = ∑_*bond* − *lengths*_1/2 *k*_*b*_(*σ* − *σ*_0_)^2^ + ∑_*bond* − *angles*_1/2 *k*_*θ*_(*θ* − *θ*_0_)^2^ + ∑_*dihedral*_(*A*[1 + *cosφ*] + *B*[1 − *cosφ*] + *C*[1 + *cos*3*φ*] + *D*[1 + *cos*(*φ* + *π*/4)]) + ∑_*non* − *bonded*_4*ϵ*_*H*_ *S*_1_[(*σ*/*r*_*ij*_)^12^ − *S*_2_(*σ*/*r*_*ij*_)^6^]. Harmonic potentials are used for bond lengths and bond angles, with *k*_*b*_ = (100*ϵ*_*h*_)/*σ* ^2^, *σ*_0_ = 3.8 Å, *k*_*θ*_ = 20*ϵ*_*h*_ *rad* ^−2^, *θ*_0_ = 105°, and *ϵ*_*h*_ = 1.25 *kcal*/*mol*. Secondary structural elements are reproduced by dihedral angles interactions that favor helical (A = 0, B = C = D = 1.2 *ϵ*_*h*_), turn (A = B = D = 0, C = 0.2*ϵ*_*h*_), or extended (A = 0.9*ϵ*_*h*_, C = 1.2 *ϵ*_*h*_, B = D = 0) backbone configurations (Fig 2). Non-bonded interactions involve all (*ij*) pairs with *j* ≥ *i* + 3, and are given by *S*_1_ = *S*_2_ = 1 for BB, $S_{1}=\frac{1}{3},S_{2}=-1$ for BL and LL, and *S*_1_ = 1, *S*_2_ = 0 for BN, LN, and NN pairs [57, 62]. In the present model rigid bond lengths described by Sorenson and Head-Gordon are replaced by harmonic interactions. Amino acids of the SsrA tag, which is covalently attached at the C-terminus of the *α*/*β* protein, are modeled to favor turn conformations. Simulations are performed at *T* ≃ 0.7 *T*_*f*_ or *T* ≃ 0.9 *T*_*f*_, where the folding temperature for this model *T*_*f*_ ≃ 260K.

### The native conformation of the *α*/*β*SP

The native state of the *α*/*β* protein is obtained using the parameters shown above. We use an annealing procedure, by heating to T = 1.35 *ϵ*_*h*_/*k*_*b*_ and cooling in decrements of 0.079 *ϵ*/*k*_*b*_ until *T* = 0.64 *ϵ*_*h*_/*k*_*b*_ is reached. Next, the structure is cooled to *T* = 0 in decrements of 0.015 *ϵ*_*h*_/*k*_*b*_ and energy minimization using a steepest descent algorithm is applied. This procedure is repeated three times, identifying the native state as the lowest energy structure. From these annealing simulations, the lowest energy structure has an energy of -31.9*ϵ*_*h*_. While this structure is slightly higher in energy compared to that found by the Head-Gordon group, a contact analysis reveals a nearly identical native structure [82].

### ClpY allostery

We describe allosteric cycles of ClpY by using the approach outlined in our earlier study [50]. Crystal structures of ATP-bound conformations of ClpY, with Protein Data Bank (PDB) ID 1DO2, are used to describe “open” pore conformation (diameter ≃ 19Å) and crystal structures of ADP-bound conformations of ClpY, with PDB ID 1DO0, are used to describe the “closed” pore conformation (diameter ≃ 8 Å) [7]. These structures are aligned to minimize their root-mean-square deviation. Sequential intra-ring transitions during the ClpY cycle are represented by motions of pairs of adjacent subunits between their open and closed conformations, so that each amino acid moves at a constant velocity between its two end locations. Computationally, allosteric transitions are modeled using the generalized constant velocity subroutine in CHARMM [81], which has been implemented to describe subunit motions to study GroEL-assisted SP folding [83]. Beads representing amino acids that belong to allosterically inactive subunits are constrained to fixed locations. Thus, a total of six moves is required to describe a full ClpY cycle between open and closed conformations. The *α*/*β*-SsrA SP is oriented along the pore axis (z-axis) on the proximal side of ClpY in the open (ATP bound) conformation, with C-terminus a distance *d*_0_ = 8 Å from the pore entrance located at z = 0 Å (Fig 1A). Resulting trajectories which bind to the loops are continued for 50 *τ* of unassisted allostery, where *τ* is the duration of a single cycle. To mimic ClpQ interactions with the partially translocated polypeptide segments, trajectories which result in partial translocation are continued for an additional 70 *τ* using an additional harmonic restraint with *k* = 0.5 kcal/(mol⋅Å^2^) and equilibrium length of 6.5 Å is applied to amino acids in the distal region ($z-{\bar{z}}_{loops}>14.5Å$).

### ClpY⋅*α*/*β*-SsrA interaction

Non-bonded intermolecular interactions between ClpY and *α*/*β*-SsrA are scaled by *λ*, *V*_*G*_*i*_, *H*_*j*__ = *λ*_*G*_*i*_, *H*_*j*__ *V*_*H*_*i*_, *H*_*j*__, where *G* = {*ClpY*, *SsrA*}, *H* = {*SsrA*, *α*/*β*} and *ij* = {*B*, *L*, *N*}. As in our previous simulations [50], we use *λ*_*SsrA*, *α*/*β*_ = *λ*_*SsrA*_*B*_, *SsrA*_*B*__ = 0.25, *λ*_*SsrA*_*L*_, *SsrA*_*L*__ = 1 to prevent SsrA from destabilizing the folded structure of the *α*/*β* SP and to reflect the random coil SsrA conformation. Hydrophobic amino acids located on the distal surface of ClpY are given an interaction *λ*_*ClpY*_*B*_, *H*_ = 2.0 to prevent translocation reversal in the absence of explicit interactions between the SP and ClpQ. Higher (lower) affinity of ClpY loops during closing (opening) transitions are described using *λ*_*ClpY*_*B*_, *H*_ = 1.5(1.0). The strength of the ClpY-SP interaction is calibrated to reproduce forces on the order of ≃100 pN. Alternatively, the interaction strength can be parametrized based on atomistic simulations of the ClpY-SP system. All other amino acids of ClpY interact using *λ*_*ClpY*, *H*_ = 1.25.

### Calculation of the fraction of native contacts

The native content of conformations of the *α*/*β* protein is determined by calculating the fraction of native contacts, $Q_{N}\left(t\right)=\frac{1}{N_{C}}\sum_{i\neq j,j\pm 1}\Theta [\eta -|r_{ij}\left(t\right)-r_{ij}^{0}|]$, where $r_{ij}^{k}\left(t\right)$ is the distance between residues *i* and *j* at time *t* and the index “0” corresponds to the native state. Native state contacts are identified using a cutoff of 8 Å for (*i*, *j*) pairs with *j* > *i* + 1. The Heaviside step function Θ(*x*) is 1 for *x* ≥ 0 and 0 for *x* < 0 and the tolerance *η* = 2 Å. The native state is characterized by *Q*_*N*_ > 0.91, as determined by fluctuations in the bulk simulations (S1 Fig).

### Calculation of characteristic time scales of unfolding and translocation

We determine the characteristic time scales using the mean first passage time [84] for unfolding or translocation: $1/\tau =\frac{1}{N_{traj}}\sum_{i=1,N_{traj}}\left(1/\tau_{i}\right)$, where *N*_*traj*_ is the total number of trajectories and *τ*_*i*_ is the first passage time for each trajectory. The first passage time for unfolding is determined using the *Q*_*N*_ < 0.91 criterion. The first passage time for the second unfolding event, the removal of the *β*4 strand from the folded structure, is computed using *Q*_*N*_ < 0.65. For translocation, the first passage time for translocation events is obtained based on propagation of SP amino acids into the distal region of ClpY.

## Supporting Information

S1 Fig Bulk equilibration of the SP. Probability density distribution of the fraction of native contacts of the *α*/*β* protein (black), of the *α*/*β*-SsrA fusion protein (red) in the bulk, and of the *α*/*β*-SsrA SP following binding to the non-allosteric ClpY pore (green).
(EPS)

S2 Fig Bulk mechanical unfolding of the SP. Force-extension curve along the N-terminal unfolding pathway in an AFM-type pulling simulation. Snapshots indicate SP conformations following unfolding events. The C-terminal region is highlighted in red and the N-terminal region in blue.
(EPS)

S3 Fig Effect of distal restraints on translocation of the *α*/*β* SP through the ClpY pore. Average contour length of the polypeptide segment translocated, as a function of time, through an allosteric (black) or non-allosteric (red) pore in the presence of distal harmonic restraints (see Methods). Simulation trajectories are initiated from the *t* = 50 *τ* configurations involving partial SP translocation effected by the allosteric pore in the absence of distal restraints.
(EPS)

S4 Fig Interaction between the SP and the I-domain in allosteric cycles of ClpY. Probability density map of interaction between the SP and the I-domain,*E*, and the radius of gyration of the SP in trajectories which (A) result in translocation and (B) do not result in translocation.
(EPS)

S5 Fig Unfolding and translocation forces. Probability density distributions of forces exerted onto the SP during unfolding and translocation in allosteric cycles of ClpY (black) and in mechanical pulling with constant velocity through a non-allosteric ClpY pore (red).
(EPS)

## References

[1] Wickner S, Maurizi MR, Gottesman S. Posttranslational Quality Control: Folding, Refolding, and Degrading Proteins. Science. 1999;286:1888–1893. 10.1126/science.286.5446.1888 10583944

[2] Hanson PI, Whiteheart SW. AAA+ proteins: have engine, will work. Nat Rev Mol Cell Biol. 2005;6:519–529. 10.1038/nrm1684 16072036

[3] Enemark EJ, Joshua-Tor L. On helicases and other motor proteins. Curr Opin Struct Biol. 2008;18:243–257. 10.1016/j.sbi.2008.01.007 18329872 PMC2396192

[4] Sauer RT, Baker TA. AAA+ Proteases: ATP-Fueled Machines of Protein Destruction. Annu Rev Biochem. 2011;80:587–612. 10.1146/annurev-biochem-060408-172623 21469952

[5] Kessel M, Maurizi MR, Kim B, Kocsis E, Trus BL, Singh SK, et al Homology in Structural Organization Between E. coli ClpAP Protease and the Eukaryotic 26 S Proteasome. J Mol Biol. 1995;250:587–594. 10.1006/jmbi.1995.0400 7623377

[6] Ogura T, Wilkinson A. AAA+ superfamily ATPases: common structure–diverse function. Genes to Cells. 2001;6:575–597. 10.1046/j.1365-2443.2001.00447.x 11473577

[7] Bochtler M, Hartmann C, Song HK, Bourenkov GP, Bartunik HD, Huber R. The structures of HslU and the ATP-dependent protease HslU-HslV. Nature. 2000;403:800–805. 10.1038/35001629 10693812

[8] Sousa MC, Trame CB, Tsuruta H, Wilbanks SM, Reddy VS, McKay DB. Crystal and Solution Structures of an HslUV Protease-Chaperone Complex. Cell. 2000;103:633–643. 11106733 10.1016/s0092-8674(00)00166-5

[9] Wang J, Song JJ, Seong IS, Franklin MC, Kamtekar S, Eom SH, et al Nucleotide-Dependent Conformational Changes in a Protease-Associated ATPase HslU. Structure. 2001;9:1107–1116. 10.1016/S0969-2126(01)00670-0 11709174

[10] Song HK, Hartmann C, Ramachandran R, Bochtler M, Behrendt R, Moroder L, et al Mutational studies on HslU and its docking mode with HslV. Proc Natl Acad Sci USA. 2000;97:14103–14108. 10.1073/pnas.250491797 11114186 PMC18878

[11] Wang J. A corrected quaternary arrangement of the peptidase HslV and ATPase HslU in a cocrystal structure. J Struct Biol. 2001;134:15–24. 10.1006/jsbi.2001.4347 11469873

[12] Guo F, Maurizi M, Esser L, Xia D. Crystal structure of ClpA, an Hsp100 chaperone and regulator of ClpAP protease. J Biol Chem. 2002;277:46743–46752. 10.1074/jbc.M207796200 12205096

[13] Kim DY, Kim KK. Crystal Structure of ClpX Molecular Chaperone from Helicobacter pylori. J Biol Chem. 2003;278:50664–50670. 10.1074/jbc.M305882200 14514695

[14] Rohrwild M, Pfeifer G, Santarius U, Muller SA, Huang HC, Engel A, et al The ATP-dependent HslVU protease from Escherichia coli is a four-ring structure resembling the proteasome. Nat Struct Mol Biol. 1997;4:133–139. 10.1038/nsb0297-133 9033594

[15] Ishikawa T, Maurizi MR, Belnap D, Steven AC. ATP-dependent proteases: Docking of components in a bacterial complex. Nature. 2000;408:667–668. 10.1038/35047165 11130060

[16] Kessel M, Wu WF, Gottesman S, Kocsis E, Steven AC, Maurizi MR. Six-fold rotational symmetry of ClpQ, the E. coli homolog of the 20S proteasome, and its ATP-dependent activator, ClpY. FEBS Lett. 1996;398:274–278. 10.1016/S0014-5793(96)01261-6 8977122

[17] Beuron F, Maurizi MR, Belnap DM, Kocsis E, Booy FP, Kessel M, et al At Sixes and Sevens: Characterization of the Symmetry Mismatch of the ClpAP Chaperone-Assisted Protease. J Struct Biol. 1998;123:248–259. 10.1006/jsbi.1998.4039 9878579

[18] Ortega J, Lee HS, Maurizi MR, Steven AC. ClpA and ClpX ATPases bind simultaneously to opposite ends of ClpP peptidase to form active hybrid complexes. J Struct Biol. 2004;146:217–226. 10.1016/j.jsb.2003.11.023 15037252

[19] Flynn JM, Neher SB, Kim Y, Sauer RT, Baker TA. Proteomic Discovery of Cellular Substrates of the ClpXP Protease Reveals Five Classes of ClpX-Recognition Signals. MolCell. 2003;11:671–683. 10.1016/s1097-2765(03)00060-1 12667450

[20] Gottesman S, Clark W, Maurizi M. The ATP-dependent Clp protease of *Escherichia coli*. Sequence of *clpA* and identification of a Clp-specific substrate. J Biol Chem. 1990;265:7886–7893. 2186030

[21] Levchenko I, Yamauchi M, Baker TA. ClpX and MuB interact with overlapping regions of Mu transposase: implications for control of the transposition pathway. Genes Dev. 1997;11:1561–1572. 10.1101/gad.11.12.1561 9203582

[22] Hoskins JR, Kim SY, Wickner S. Substrate Recognition by the ClpA Chaperone Component of ClpAP Protease. J Biol Chem. 2000;275:35361–35367. 10.1074/jbc.M006288200 10952988

[23] Park E, Rho YM, Koh O, Ahn SW, Seong IS, Song JJ, et al Role of the GYVG Pore Motif of HslU ATPase in Protein Unfolding and Translocation for Degradation by HslV Peptidase. J Biol Chem. 2005;280:22892–22898. 10.1074/jbc.M500035200 15849200

[24] Yamada-Inagawa T, Okuno T, Karata K, Yamanaka K, Ogura T. Conserved Pore Residues in the AAA Protease FtsH Are Important for Proteolysis and Its Coupling to ATP Hydrolysis. J Biol Chem. 2003;278:50182–50187. 10.1074/jbc.M308327200 14514680

[25] Martin A, Baker TA, Sauer RT. Pore loops of the AAA+ ClpX machine grip substrates to drive translocation and unfolding. Nat Struct Mol Biol. 2008;15:1147–1151. 10.1038/nsmb.1503 18931677 PMC2610342

[26] Lee C, Schwartz MP, Prakash S, Iwakura M, Matouschek A. ATP-Dependent Proteases Degrade Their Substrates by Processively Unraveling Them from the Degradation Signal. Mol Cell. 2001;7:627–637. 10.1016/S1097-2765(01)00209-X 11463387

[27] Li H, Carrión-Vázquez M, Oberhauser AF, Marszalek PE, Fernandez JM. Point mutations alter the mechanical stability of immunoglobulin modules. Nat Struct Biol. 2000;7:1117–1120. 10.1038/81964 11101892

[28] Kenniston JA, Baker TA, Fernandez JM, Sauer RT. Linkage between ATP Consumption and Mechanical Unfolding during the Protein Processing Reactions of an AAA+ Degradation Machine. Cell. 2003;114:511–520. 10.1016/S0092-8674(03)00612-3 12941278

[29] Huang YM, Bystroff C. Complementation and reconstitution of fluorescence from circularly permuted and truncated green fluorescent protein. Biochemistry. 2009;48:929–940. 10.1021/bi802027g 19140681 PMC4651016

[30] Nager AR, Baker TA, Sauer RT. Stepwise Unfolding of a *β* Barrel Protein by the AAA+ ClpXP Protease. J Mol Biol. 2011;413:4–16. 10.1016/j.jmb.2011.07.041 21821046 PMC3184388

[31] Shin Y, Davis JH, Brau RR, Martin A, Kenniston JA, Baker TA, et al Single-molecule denaturation and degradation of proteins by the AAA+ ClpXP protease. PNAS. 2009;106:19340–19345. 10.1073/pnas.0910484106 19892734 PMC2773733

[32] Aubin-Tam ME, Olivares A, Sauer R, Baker T, Lang M. Single-Molecule Protein Unfolding and Translocation by an ATP-Fueled Proteolytic Machine. Cell. 2011;145:257–267. 10.1016/j.cell.2011.03.036 21496645 PMC3108460

[33] Maillard R, Chistol G, Sen M, Righini M, Tan J, Kaiser CM, et al ClpX(P) Generates Mechanical Force to Unfold and Translocate Its Protein Substrates. Cell. 2011;145:459–469. 10.1016/j.cell.2011.04.010 21529717 PMC3686100

[34] Sen M, Maillard R, Nyquist K, Rodriguez-Aliaga P, Pressé S, Martin A, et al The ClpXP Protease Unfolds Substrates Using a Constant Rate of Pulling but Different Gears. Cell. 2013;155:636–646. 10.1016/j.cell.2013.09.022 24243020 PMC3901371

[35] Cordova J, Olivares A, Shin Y, Stinson B, Calmat S, Schmitz K, et al Stochastic but Highly Coordinated Protein Unfolding and Translocation by the ClpXP Proteolytic Machine. Cell. 2014;158:647–658. 10.1016/j.cell.2014.05.043 25083874 PMC4134808

[36] Olivares AO, Nager AR, Iosefson O, Sauer RT, Baker TA. Mechanochemical basis of protein degradation by a double-ring AAA+ machine. Nat Struct Mol Biol. 2014;21:871–875. 10.1038/nsmb.2885 25195048 PMC4190165

[37] Martin A, Baker TA, Sauer RT. Protein unfolding by a AAA+ protease is dependent on ATP-hydrolysis rates and substrate energy landscapes. Nat Struct Mol Biol. 2008;15:139–145. 10.1038/nsmb.1380 18223658

[38] Chistol G, Liu S, Hetherington CL, Moffitt JR, Grimes S, Jardine PJ, et al High degree of coordination and division of labor among subunits in a homomeric ring ATPase. Cell. 2012;151:1017–1028. 10.1016/j.cell.2012.10.031 23178121 PMC3652982

[39] Maurizi M, Stan G. ClpX Shifts into High Gear to Unfold Stable Proteins. Cell. 2013;155:502–504. 10.1016/j.cell.2013.10.007 24243009

[40] Huang L, Kirmizialtin S, Makarov DE. Computer simulations of the translocation and unfolding of a protein pulled mechanically through a pore. J Chem Phys. 2005;123:124903 10.1063/1.2008231 16392523

[41] West DK, Brockwell DJ, Paci E. Prediction of the Translocation Kinetics of a Protein from Its Mechanical Properties. Biophys J. 2006;91:L51–53. 10.1529/biophysj.106.089490 16815903 PMC1544310

[42] Szymczak P, Janovjak H. Periodic forces trigger a complex mechanical response in ubiquitin. J Mol Biol. 2009;390:443–456. 10.1016/j.jmb.2009.04.071 19426737

[43] Wojciechowski M, Szymczak P, Carrión-Vázquez M, Cieplak M. Protein Unfolding by Biological Unfoldases: Insights from Modeling. Biophys J. 2014;107:1661–1668. 10.1016/j.bpj.2014.07.035 25296319 PMC4190598

[44] Feng G, Lu H. Computer simulation of I27 translocation through ClpY reveals a critical role of protein mechanical strength and local stability. In: Proceedings of the 29th Annual International Conference of the IEEE EMBS Cité Internationale, Lyon, France; 2007. 10.1109/IEMBS.2007.4352515 18002181

[45] Tonddast-Navaei S, Stan G. Mechanism of Transient Binding and Release of Substrate Protein during the Allosteric Cycle of the p97 Nanomachine. J Am Chem Soc. 2013;135:14627–14636. 10.1021/ja404051b 24007343

[46] Koga N, Kameda T, Okazaki K, Takada S. Paddling mechanism for the substrate translocation by AAA+ motor revealed by multiscale molecular simulations. Proc Natl Acad Sci USA. 2009;106:18237–18242. 10.1073/pnas.0904756106 19828442 PMC2775326

[47] Yoshimoto K, Arora K, Brooks CL III. Hexameric Helicase Deconstructed: Interplay of Conformational Changes and Substrate Coupling. Biophys J. 2010;98:1449–1457. 10.1016/j.bpj.2009.12.4315 20409463 PMC2856183

[48] Jana B, Morcos F, Onuchic JN. From structure to function: the convergence of structure based models and co-evolutionary information. Phys Chem Chem Phys. 2013;16:6496–6507. 10.1039/c3cp55275f 24603809

[49] Ma W, Schulten K. Mechanism of Substrate Translocation by a Ring-Shaped ATPase Motor at Millisecond Resolution. J Am Chem Soc. 2015;137:3031–3040. 10.1021/ja512605w 25646698 PMC4393844

[50] Kravats A, Jayasinghe M, Stan G. Unfolding and translocation pathway of substrate protein controlled by structure in repetitive allosteric cycles of the ClpY ATPase. Proc Natl Acad Sci USA. 2011;108:2234–2239. 10.1073/pnas.1014278108 21266546 PMC3038749

[51] Kravats AN, Tonddast-Navaei S, Bucher RJ, Stan G. Asymmetric processing of a substrate protein in sequential allosteric cycles of AAA+ nanomachines. J Chem Phys. 2013;139:121921 10.1063/1.4817410 24089733

[52] Mickler M, Dima RI, Dietz H, Hyeon C, Thirumalai D, Rief M. Revealing the bifurcation in the unfolding pathways of GFP using single molecule experiments and simulations. Proc Natl Acad Sci USA. 2007;104:20268–20273. 10.1073/pnas.0705458104 18079292 PMC2154420

[53] Graham TGW, Best RB. Force-Induced Change in Protein Unfolding Mechanism: Discrete or Continuous Switch? J Phys Chem B. 2011;115:1546–1561. 10.1021/jp110738m 21271708

[54] Li YD, Lamour G, Gsponer J, Zheng P, Li H. The Molecular Mechanism Underlying Mechanical Anisotropy of the Protein GB1. Biophys J. 2012;103:2361–2368. 10.1016/j.bpj.2012.10.035 23283235 PMC3514518

[55] Valbuena A, Oroz J, Hervás R, Vera AM, Rodríguez D, Menéndez M, et al On the remarkable mechanostability of scaffoldins and the mechanical clamp motif. Proc Natl Acad Sci USA. 2009;106:13791–13796. 10.1073/pnas.0813093106 19666489 PMC2719556

[56] Best RB, Hummer G, Eaton WA. Native contacts determine protein folding mechanisms in atomistic simulations. Proc Natl Acad Sci USA. 2013;110:17874–17879. 10.1073/pnas.1311599110 24128758 PMC3816414

[57] Sorenson JM, Head-Gordon T. Matching Simulation and Experiment: A New Simplified Model for Simulating Protein Folding. J Comput Biol. 2000;7:469–481. 10.1089/106652700750050899 11108474

[58] Humphrey W, Dalke A, Schulten K. VMD—Visual Molecular Dynamics. J. Mol. Graphics 1996;14:33–38. 10.1016/0263-7855(96)00018-5 8744570

[59] Brockwell DJ, Beddard GS, Paci E, West DK, Olmsted PD, Smith DA, et al Mechanically unfolding the small, topologically simple protein L. Biophys J. 2005;89:506–519. 10.1529/biophysj.105.061465 15863479 PMC1366550

[60] de Graff AMR, Shannon G, Farrell DW, Williams PM, Thorpe MF. Protein unfolding under force: crack propagation in a network. Biophys J. 2011;101:736–744. 10.1016/j.bpj.2011.05.072 21806942 PMC3145275

[61] West DK, Olmsted PD, Paci E. Mechanical unfolding revisited through a simple but realistic model. J Chem Phys. 2006;124 10.1063/1.2185100 16674267

[62] Brown S, Fawzi NJ, Head-Gordon T. Coarse-grained sequences for protein folding and design. Proc Natl Acad Sci USA. 2003;100:10712–10717. 10.1073/pnas.1931882100 12963815 PMC196869

[63] Hersch GL, Burton RE, Bolon DN, Baker TA, Sauer RT. Asymmetric Interactions of ATP with the AAA+ ClpX_6_ Unfoldase: Allosteric Control of a Protein Machine. Cell. 2005;121:1017–1027. 10.1016/j.cell.2005.05.024 15989952

[64] Yakamavich JA, Baker TA, Sauer RT. Asymmetric Nucleotide Transactions of the HslUV Protease. J Mol Biol. 2008;380:946–957. 10.1016/j.jmb.2008.05.070 18582897 PMC2517146

[65] Smith DM, Fraga H, Reis C, Kafri G, Goldberg AL. ATP Binds to Proteasomal ATPases in Pairs with Distinct Functional Effects, Implying an Ordered Reaction Cycle. Cell. 2011;144:526–538. 10.1016/j.cell.2011.02.005 21335235 PMC3063399

[66] Iosefson O, Nager AR, Baker TA, Sauer RT. Coordinated gripping of substrate by subunits of a AAA + proteolytic machine. 2015;11:201–206. 10.1038/nchembio.1732 PMC4333055 25599533

[67] Martin A, Baker TA, Sauer RT. Rebuilt AAA + motors reveal operating principles for ATP-fuelled machines. Nature. 2005;437:1115–1120. 10.1038/nature04031 16237435

[68] Sundar S, Baker TA, Sauer RT. The I domain of the AAA+ HsIUV protease coordinates substrate binding, ATP hydrolysis, and protein degradation. Prot Sci. 2012;21:188–198. 10.1002/pro.2001 PMC3324763 22102327

[69] Too PHM, Erales J, Simen JD, Marjanovic A, Coffino P. Slippery substrates impair function of a bacterial protease ATPase by unbalancing translocation versus exit. J Biol Chem. 2013;288:13243–57. 10.1074/jbc.M113.452524 23530043 PMC3650364

[70] Tian L, Holmgren RA, Matouschek A. A conserved processing mechanism regulates the activity of transcription factors Cubitus interruptus and NF-*κ*B. Nat Struct Mol Biol. 2005;12:1045–1053. 10.1038/nsmb1018 16299518

[71] Hoyt MA, Zich J, Takeuchi J, Zhang M, Govaerts C, Coffino P. Glycine-alanine repeats impair proper substrate unfolding by the proteasome. EMBO J. 2006;25:1720–1729. 10.1038/sj.emboj.7601058 16601692 PMC1440830

[72] Vass RH, Chien P. Critical clamp loader processing by an essential AAA+ protease in *Caulobacter crescentus* . Proc Natl Acad Sci USA. 2013;110:18138–18143. 10.1073/pnas.1311302110 24145408 PMC3831445

[73] Glynn SE, Nager AR, Baker TA, Sauer RT. Dynamic and static components power unfolding in topologically closed rings of a AAA+ proteolytic machine. Nat Struct Mol Biol. 2012;19:616–622. 10.1038/nsmb.2288 22562135 PMC3372766

[74] Burton RE, Siddiqui SM, Kim YI, Baker TA, Sauer RT. Effects of protein stability and structure on substrate processing by the ClpXP unfolding and degradation machine. EMBO J. 2001;20:3092–3100. 10.1093/emboj/20.12.3092 11406586 PMC150209

[75] Hoskins JR, Yanagihara K, Mizuuchi K, Wickner S. ClpAP and ClpXP degrade proteins with tags located in the interior of the primary sequence. Proc Natl Acad Sci USA. 2002;17:11037–11042. 10.1073/pnas.172378899 PMC123206 12177439

[76] Huang L, Makarov DE. Translocation of a knotted polypeptide through a pore. J Chem Phys. 2008;129:121107 10.1063/1.2968554 19044999

[77] Rosa A, Ventra MD, Micheletti C. Topological Jamming of Spontaneously Knotted Polyelectrolyte Chains Driven Through a Nanopore. Phys Rev Lett. 2012;109:118301 10.1103/PhysRevLett.109.118301 23005684

[78] Szymczak P. Translocation of knotted proteins through a pore. Eur Phys J Special Topics. 2014;223:1805–1812. 10.1140/epjst/e2014-02227-6

[79] Honeycutt JD, Thirumalai D. The nature of folded states of globular—proteins. Biopolymers. 1992;32:695–709. 10.1002/bip.360320610 1643270

[80] Guo Z, Thirumalai D. Kinetics and Thermodynamics of Folding of a *de Novo* Designed Four—helix Bundle Protein. J Mol Biol. 1996;263:323–343. 10.1006/jmbi.1996.0578 8913310

[81] Brooks BR, Bruccoleri RE, Olafson BD, States DJ, Swaminathan S, Karplus M. CHARMM: A Program for Macromolecular Energy, Minimization and Dynamics Calculations. J Comp Chem. 1983;4:187–217. 10.1002/jcc.540040211

[82] Brown S, Head-Gordon T. Intermediates and the folding of proteins L and G. Prot Sci. 2004;13:958–970. 10.1110/ps.03316004 PMC2280051 15044729

[83] Stan G, Lorimer GH, Thirumalai D, Brooks BR. Coupling between allosteric transitions in GroEL and assisted folding of a substrate protein. Proc Natl Acad Sci USA. 2007;104:8803–8808. 10.1073/pnas.0700607104 17496143 PMC1885583

[84] Klimov DK, Newfield D, Thirumalai D. Simulations of *β*-hairpin folding confined to spherical pores using distributed computing. Proc Natl Acad Sci USA. 2002;99:8019–8024. 10.1073/pnas.072220699 12060748 PMC123013

